# A therapeutic hepatitis B mRNA vaccine with strong immunogenicity and persistent virological suppression

**DOI:** 10.1038/s41541-024-00813-3

**Published:** 2024-02-03

**Authors:** Huajun Zhao, Xianyu Shao, Yating Yu, Lulu Huang, Narh Philip Amor, Kun Guo, Changzhen Weng, Weijun Zhao, Ailu Yang, Jiesen Hu, Hongbao Yang, Zhenguang Liu, Qiuju Han, Leilei Shi, Shiyu Sun, Jian Zhang, Ang Lin, Yong Yang

**Affiliations:** 1 Institute of Immunopharmaceutical Sciences, Key Laboratory of Chemical Biology, School of Pharmaceutical Sciences, Shandong University, Jinan, Shandong China; 2 Vaccine Center, School of Basic Medicine and Clinical Pharmacy, China Pharmaceutical University, Nanjing, China; 3 Center for New Drug Safety Evaluation and Research, China Pharmaceutical University, Nanjing, China; 4 Firestone Biotechnologies, Shanghai, China; 5 College of Veterinary Medicine, Nanjing Agricultural University, Nanjing, China; 6 Precision Research Center for Refractory Diseases in Shanghai General Hospital, Shanghai Jiao Tong University, Shanghai, China; 7 Key Laboratory of Infection and Immunity, Institute of Biophysics, Chinese Academy of Sciences, Beijing, China; 8 School of Pharmacy, Xuzhou Medical University, Xuzhou, 221004 Jiangsu PR China

**Keywords:** RNA vaccines, Hepatitis B, RNA vaccines

## Abstract

Here we report on the development and comprehensive evaluations of an mRNA vaccine for chronic hepatitis B (CHB) treatment. In two different HBV carrier mouse models generated by viral vector-mediated HBV transfection (pAAV-HBV1.2 and rAAV8-HBV1.3), this vaccine demonstrates sufficient and persistent virological suppression, and robust immunogenicity in terms of induction of strong innate immune activation, high-level virus-specific antibodies, memory B cells and T cells. mRNA platform therefore holds prospects for therapeutic vaccine development to combat CHB.

CHB is one major cause of liver fibrosis, cirrhosis, and hepatocellular carcinoma and poses a major public health threat^1^. Current CHB treatments such as nucleoside/nucleotide- and interferon-alpha (IFN-α)-based therapy, were unable to achieve sufficient viral clearance^2,3^. Multiple approaches to CHB therapeutic vaccines have been practiced intensively, including recombinant protein-based subunit, adenoviral vectored, and DNA-based vaccines, but yet achieved efficient seroclearance of HBsAg and seroconversion of anti-HBs antibody (Ab)^4–6^. mRNA vaccines that contain antigen-encoding mRNAs encapsulated into for example lipid nanoparticle (LNP) have shown superior immunogenicity in eliciting both Ab and cellular immune responses over other types of vaccines, and are also endowed with strong intrinsic adjuvant property to activate innate immune compartment^7^. Several mRNA-based prophylactic or therapeutic vaccines for infections, malignancies or other diseases are currently being evaluated in clinical trials^8–11^. This platform also holds prospects for the development of therapeutic CHB vaccine that is expected to elicit potent anti-viral immunity mediating efficient virological suppression.

Recently, we reported a proprietary artificial intelligence-based algorithm that designs mRNA with optimal folding stability and codon usage that together contribute to a high translation efficiency^12^. Using this algorithm, an mRNA vaccine for CHB treatment was developed, which is composed of Hepatitis B surface antigen (HBsAg)-encoding mRNAs encapsulated into an ionizable lipid-based LNP through a well-established microfluidic system^13^. The mRNAs were modified with N1-Methyl-pseudouridine and showed efficient protein expression reaching a mean level of 980.6 mIU/ml upon transfection into HEK-293T cells (Supplementary Fig. 1a). Vaccine formulations were well characterized which demonstrated particle diameters of 96.3 ± 2.16 nm with average zeta potential of −1.92 mV and polymer dispersity index (PDI) below 0.2. In addition, cells incubated with escalating concentrations of mRNA vaccines for 24 h maintained high viability, which suggested a limited cytotoxicity of vaccine (Supplementary Fig. 1b).

Therapeutic efficacy of the mRNA vaccine was first evaluated in pAAV-HBV1.2-transduced HBV-carrier mice. This model shows systemic immune tolerance and long-lasting HBV viremia that largely resemble asymptomatic chronic HBV-infected individuals and has therefore been widely used in the study of CHB immunotherapy^14,15^. HBV-carrier mice were administered intramuscularly (i.m.) with three doses of 5 μg or 10 μg mRNA vaccines at a 1-week interval (Fig. 1a). Compared to PBS-treated mice showing a high serum HBsAg level, mRNA vaccine-treated mice demonstrated a rapid decline of serum HBsAg, which was even undetectable 7 days after the 3^rd^ dose (Fig. 1b). Clinical management of CHB remains challenging largely due to HBV recurrence and the failure to achieve seroconversion of anti-HBs Abs^2,3^. Magnitude of anti-HBs Ab response was therefore evaluated longitudinally. Three doses of HBV mRNA vaccines elicited robust levels of anti-HBs Abs reaching a mean titer of 3624.0 mIU/ml and 4804.6 mIU/ml in the 5 μg and 10 μg vaccine groups at day 59, respectively (Fig. 1c).

**Fig. 1:**
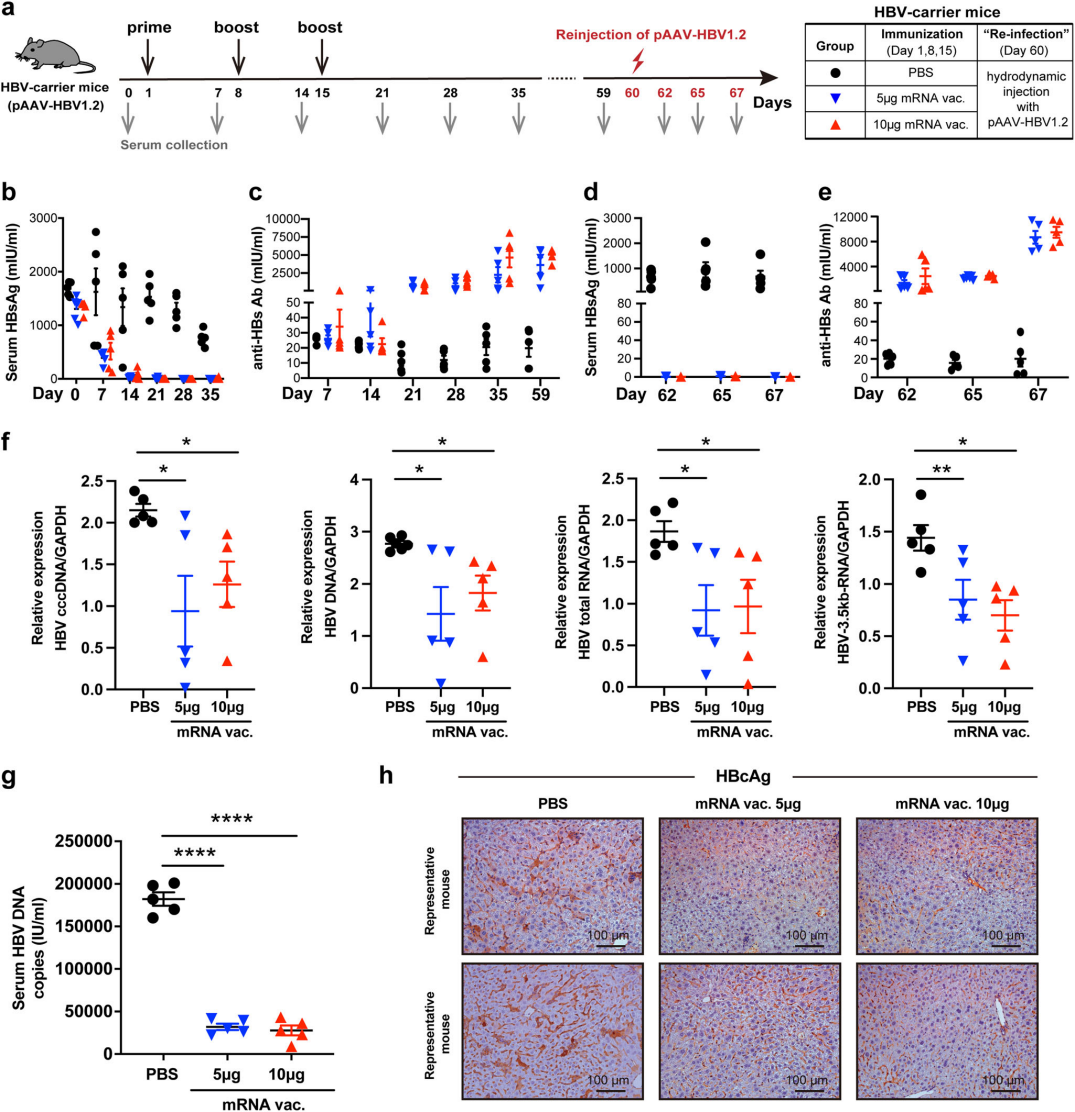
HBV mRNA vaccine induced efficient viral suppression and achieved robust seroconversion in pAAV-HBV1.2 mice. **a** Study design. pAAV-HBV1.2-transduced HBV-carrier mice (*n* = 5/group) were immunized i.m. with 5 μg or 10 μg HBV mRNA vaccines three times at a 1-week interval. HBV-carrier mice administered with PBS were used as control. At day 60, mice were hydrodynamically re-injected with 8 μg pAAV-HBV1.2 plasmids. Sera samples were collected at the indicated time points. **b**, **d** Levels of serum HBsAg and **c**, **e** anti-HBs Abs were measured at the indicated time points. **f** Seven days after viral re-exposure, intrahepatic HBV cccDNA, total DNA, total RNA and 3.5-kb RNA were analyzed. **g** Seven days after viral re-exposure, serum HBV DNA was quantified. **h** Seven days after viral re-exposure, HBcAg expression in liver tissues was determined by IHC staining (magnification: ×200; scale bar: 100 µm). GAPDH, glyceraldehyde 3-phosphate dehydrogenase; SEM, standard error of the mean. An unpaired, two-tailed Student’s *t* test was used for statistical analysis. Data are shown as Mean ± SEM. *p ≤ 0.05, **p ≤ 0.01, ****p ≤ 0.0001.

To evaluate whether the mRNA vaccine could induce sustained protective responses against viral re-exposure, the mice were re-injected with pAAV-HBV1.2 plasmids on day 60 mimicking viral challenge. As expected, PBS-treated mice still demonstrated a high level of serum HBsAg. While all vaccinated mice were fully protected against viral re-exposure showing no detectable level of serum HBsAg (Fig. 1d), which was accompanied by further elevated anti-HBs Ab titers (Fig. 1e). Moreover, copies of intrahepatic HBV cccDNA, total DNA, total RNA, 3.5 kb RNA (Fig. 1f) and serum HBV DNA (Fig. 1g) were clearly reduced in vaccinated mice and the expression of HBV core antigen (HBcAg) in liver tissues was largely decreased (Fig. 1h). Since AAV-transduced-HBV-carrier mice cannot produce significant amounts of cccDNA, the anti-HBV effect on eliminating HBV cccDNA should be further evaluated in specific animal models, such as rc-cccDNA mouse model^16^ or human liver chimeric mouse model^17^.

Efficacy of the mRNA vaccine was next compared side-by-side with front-line therapeutic Entecavir (ETV). Compared to the rapid anti-HBs Ab production and serum HBsAg clearance induced by mRNA vaccine, Entecavir administered via oral gavage for consecutive 15 days showed no ability in inducing anti-HBs Abs or eliminating serum HBsAg (Supplementary Fig. 2b, c), which was in line with previous studies^18^. However, serum HBV DNA copies were significantly reduced in mRNA vaccinated or ETV-treated HBV-carrier mice (Supplementary Fig. 2d). Considering that viral clearance can be mediated through vaccine-induced cytotoxic immune responses potentially causing liver damage, serum alanine transaminase (ALT) and aspartate aminotransferase (AST) levels were monitored longitudinally (Supplementary Fig 3a, b). A transient elevation of serum ALT was observed in a few animals 7 days post the 1^st^ and 2^nd^ vaccination. While serum AST remained at normal levels in all treated mice during the period of treatment. In addition, histopathological analysis revealed that there was no hepatotoxicity or liver injury induced upon the 3-dose vaccine treatment (Supplementary Fig. 3c). These suggested that the mRNA vaccine may potentially function through a non-cytotoxic mechanism to eliminate virus. Previously, we and others showed that IFN-γ-producing HBV-specific CD8^+^ T cells could mediate HBV clearance via a non-cytotoxic mechanism without causing liver damage^19,20^. Since both cytotoxic and non-cytotoxic immune responses to HBV are important to eliminate virus, the underlying mechanisms of action of the mRNA vaccine would merit further investigation.

Therapeutic efficacy of the mRNA vaccine was further evaluated in rAAV8-HBV1.3-transduced HBV-carrier mouse model (Fig. 2a), which shows more efficient and homogeneous HBV transduction than the aforementioned pAAV-HBV1.2 mice model^21^. As validated in our study, rAAV8-HBV1.3-transduced mice showed persistent HBV surface antigenemia lasting for more than 200 days (Supplementary Fig. 4) and a typical immunotolerant status represented by high frequencies of hepatic CD4^+^CD25^+^ Foxp3^+^ T cells (Supplementary Fig. 4b), elevated expression of inhibitory immune-checkpoint molecules (PD-1, LAG-3, and TIM-3), and impaired cytokine production by CD4^+^ T and CD8^+^ T cells upon stimulation (Supplementary Fig. 4c, d). All these features well mimic the immunotolerant state of human chronic HBV carriers^21^. Upon three doses of vaccination, HBV1.3-carrier mice demonstrated a rapid and sufficient serum HBsAg clearance, and the virological suppression was maintained for at least 208 days within the period of observation (Fig. 2b). Furthermore, serum HBeAg level was remarkably reduced in both two groups of vaccinated mice (Fig. 2c). Serum HBV DNA copies were significantly reduced in mice receiving 10 μg mRNA vaccine and showed a trend of decrease in the 5 μg dosing group (Fig. 2d). Notably, majority of the 10 μg mRNA vaccine-treated mice still presented high levels of anti-HBs Abs when detected 208 days after treatment initiation (Fig. 2e). Since serum HBsAg was sufficiently cleared, but serum HBV DNA was still present which implied that viral replication may still remain active at a very low level although was largely restricted. The mRNA vaccine-elicited anti-viral immunity remains to be further assessed for the efficacy to control viral genome transcription using more suitable animal models.

**Fig. 2:**
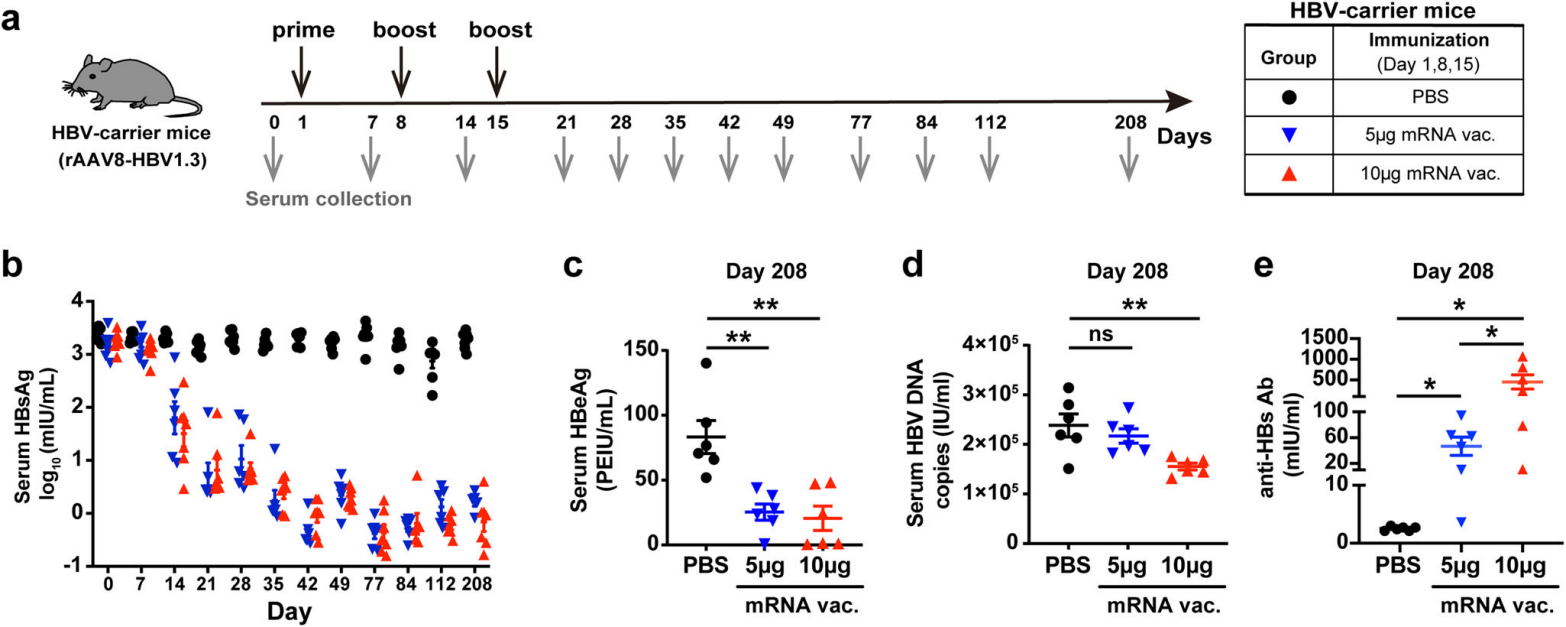
HBV mRNA vaccine induced efficient and sustained viral suppression and achieved robust seroconversion in rAAV8-HBV1.3 mice. **a** Study design. rAAV-HBV1.3-transduced HBV-carrier mice (*n* = 6/group) were immunized i.m. with 5 μg or 10 μg HBV mRNA vaccines three times at a 1-week interval. HBV-carrier mice administered with PBS were used as control. Sera samples were collected longitudinally. **b** Levels of serum HBsAg were measured at the indicated time points. 208 days after treatment start, levels of serum HBeAg (**c**), serum HBV DNA copies (**d**), and anti-HBs Abs (**e**) were measured. An unpaired, two-tailed Student’s *t* test was used for statistical analysis. Data are shown as Mean ± SEM. *p ≤ 0.05, **p ≤ 0.01. PEIU represents Paul-Ehrlich-Institute Unit.

To further validate the potency and superiority of the mRNA vaccine, we next performed a side-by-side comparison with our recently reported recombinant CHB therapeutic vaccines (Sim+rHBV)^22^ in rAAV8-HBV1.3 mice. Three doses of Sim+rHBV vaccine showed a moderate ability in viral clearance but much less efficient than the mRNA vaccine (Supplementary Fig. 5). Seven days post the 3^rd^ dose, serum HBsAg levels in mice receiving 5 μg mRNA vaccine, 10 μg mRNA vaccine and Sim+rHBV vaccine were present at a mean value of 15.8 mIU/ml, 16.0 mIU/ml and 166.0 mIU/ml, respectively. In addition, evidence from other groups testing their in-house developed therapeutic CHB vaccines using same animal models indirectly supported the superior therapeutic effect of mRNA vaccine^23,24^.

An optimal therapeutic vaccine for CHB treatment should have the potentials to trigger efficient innate immune activation leading to robust antigen presentation and the resulting generation of HBV-specific cellular immunity^12^. To this end, we studied the innate immune responses induced at early time point (12 h) after prime immunization in rAAV8-HBV1.3 mice and observed an increased infiltration of dendritic cell (DC) subsets (CD8α^+^ cDC1s, CD103^+^ cDC1s and CD11b^+^ cDC2s) and macrophages into spleen, accompanied by potent cell maturation (Supplementary Fig. 6a–e). Other innate immune cell subsets including monocytes and neutrophils (Supplementary Fig. 6f–h) also showed a phenotypic maturation, indicated by elevated expression of CD80 and CD86. T cell exhaustion is a hallmark of CHB infection and the restored HBV-specific CD8^+^ T cell function has been widely-accepted to be predictive for the efficacy of therapeutic vaccines for CHB^25^. We therefore assessed whether the therapeutic mRNA vaccination could promote HBV-specific T cell responses and break the exhaustion. In rAAV8-HBV1.3 mice, three doses of mRNA vaccines induced robust levels of Th1-biased CD4^+^ and CD8^+^ T cells producing IFN-γ or IL-2 favoring viral elimination (Supplementary Fig. 7a–c). In addition, frequencies of HBsAg-specific memory B cells (MBCs) in spleens were obviously elevated upon vaccination (Supplementary Fig. 7d) which was associated with the strong seroconversion and long-term protection as observed earlier in this study (Supplementary Fig. 2).

Collectively, we reported an HBV mRNA vaccine candidate with potent therapeutic efficacy and strong immunogenicity. Three doses of HBV mRNA vaccines could efficiently and persistently eliminate HBV and achieve a long-term seroconversion of anti-HBs Ab, and most importantly showed full protection against subsequent viral re-exposure. The strong innate immune activation and generation of robust functional HBV-specific T cells and MBCs by the mRNA vaccine may hold prospects for functional cure of CHB and prevention of HBV recurrence. However, further in-depth assessment of the mRNA vaccine would be needed to evaluate the effects on restricting viral replication at genomic levels especially the potentiality to eliminate HBV cccDNA pool. In addition, synergistic efficacy of the mRNA vaccine in combinatorial use with other types of CHB therapeutics merits further investigation.

## Methods

### Ethics, animals, treatments

C57BL/6J mice (5-6 weeks old, male) were purchased from Beijing HFK Bioscience Co. Ltd. (Beijing, China). HBV-carrier mouse models were generated either through hydrodynamic injection of a volume of saline (equivalent to 10% body weight) containing 8 μg pAAV-HBV 1.2 plasmid (kindly provided by Pei-Jer Chen; National Taiwan University College of Medicine, Taipei, Taiwan) or intravenous injection of 1 × 10^10^ vector genome equivalent of rAAV8-HBV1.3, as previously described^21,23^. Serum HBsAg levels were measured 6 weeks after the hydrodynamic or intravenous injection, and mice with serum HBsAg levels >500 mIU/mL were defined as HBV-carrier mice and used for subsequent in-vivo experiments. HBV-carrier mice were randomly allocated to different groups and were i.m. injected with three doses of 5 μg or 10 μg mRNA vaccines at a one-week interval. HBV-carrier mice administered with PBS were used as control. In some experiments, HBV-carrier mice were treated with Entecavir (50 μg/kg, Selleck Chem) via oral gavage using curved feeding needles (18-gauge, 2-inches) for 15 days consecutively or were immunized with a recombinant therapeutic vaccine (Sim+rHBV)^21^ containing 2 μg rHBVvac adjuvanted with 100 μg simvastatin. Sera samples were collected at different time points and were stored at −80 °C for further use. Mice were euthanized by inhalation of CO_2_ prior to necropsy. For the evaluation of long-term protective response, the immunized HBV-carrier mice were hydrodynamically re-injected with 8 μg pAAV-HBV1.2 plasmids on day 60 after treatment initiation. All animal experiments were performed in accordance with the Guidelines for the Care and Use of Laboratory Animals and the Ethical Committee of Shandong University and using protocols approved by the Institutional Animal Care and Use Committee of Shandong University (approval number: 20023).

### mRNA vaccine preparation

mRNAs encoding for HBsAg (NCBI accession number: YP_009173871) were synthesized by T7 polymerase-mediated in vitro transcription (IVT) based on a linearized DNA template (pUC57-GW-Kan) containing codon-optimized HBsAg gene flanked with 5’ and 3’ untranslated regions (UTRs) and a 100 nt poly-A tail. During IVT procedure, mRNAs were modified with N1-Methyl-pseudouridine (Synthgene) and capped using CleanCap Reagent (TriLink). After this, IVT products were purified with Monarch RNA purification columns (NEW ENGLAND BioLabs Inc. MA, USA) and resuspended in a TE buffer at a desired concentration. For mRNA encapsulation into LNP, lipid components were dissolved in ethanol at molar ratios of 50:10:38.5:1.5 (ionizable lipid: DSPC: cholesterol: DMG-PEG2000). The ionizable lipid (YX-02) was designed and has been patented by Firestone Biotechnologies. The lipid cocktail was mixed with mRNAs dissolved in 10 mM citrate buffer (pH4.0) at an N/P ratio of 5.3 :1 and a volume ratio of 3: 1 using a microfluidic-based equipment (INano^TM^L from Micro&Nano Biologics) at a total flow rate of 12 mL/min. Formulations were diluted with PBS and ultrafiltrated using 50-kDa Amicon ultracentrifugal filters. Vaccine formulation was characterized for particle diameter, polymer dispersity index (PDI) and zeta potentials using NanoBrook Omni ZetaPlus (Brookhaven Instruments).

### Evaluation of mRNA translation in vitro

Human embryonic kidney (HEK) 293 T cells were cultured in high-glucose Dulbecco’s Modified Eagle Medium (DMEM, BIOIND) supplemented with 10% fetal bovine serum (FBS, BIOIND) and 1% penicillin-streptomycin (NCM Biotech). 5 ×10^5^ HEK293T cells were seeded into 6-well plates and were transfected with 2 μg HBsAg-encoding mRNAs using jetMessegner® transfection reagent (Polyplus-transfection^®^) according to the instructions. Cells were harvested 48 hours later and were lysed using RIPA lysis buffer (Beyotime). Levels of HBsAg in cell lysates were quantified using HBsAg Quantification Kit (Autobio).

### Cytotoxicity of HBV mRNA vaccine

HEK-293T or AML12 cells were seeded into 96-well plates at a density of 8 ×10^3^ cells per well suspended in 100 μL DMEM medium. Cells were incubated with or without escalating concentrations of HBV mRNA vaccines (50 μg/ml, 100 μg/ml, 200 μg/ml, 400 μg/ml, 800 μg/ml, 1600μg/ml, 3200 μg/ml) for 24 hours. Cell Counting Kit-8 (CCK-8) was used to determine cytotoxicity according to the manuals. Briefly, CCK-8 reagent was added into cell culture prior to incubation at 37 °C for 1 hour. After this, absorbance of mixture was read at 450 nm wavelength using CMax Plus microplate reader (Molecular Device). Cell viability was calculated using formula: Cell viability (%) = (OD1–OD3)/(OD2–OD3) × 100%. OD1: cells treated with mRNA vaccines; OD2: cells cultured with medium alone; OD3: DMEM medium only.

### Quantification of serum HBsAg and HBeAg

Levels of serum HBsAg and HBeAg were quantified using Chemiluminescence Immunoassay (CLIA) Commercialized Kits according to the manuals (Autobio). Briefly, undiluted sera (50 μL) were added into the wells followed by incubation with detection reagent (50 μL) at 37 °C for 60 min. After washing, chemiluminescent substrates (50 μL) were added into the mixture and incubated at room temperature (RT) for 10 min in the dark. Plates were read using Synergy 2 Multi-Mode Microplate Reader (BioTek, Vermont).

### Determination of anti-HBs IgG titer

Titers of serum anti-HBs Abs were determined using commercialized ELISA kit (Wantai Bio-pharm) according to instruction. Briefly, undiluted sera (50 μL) were added into wells pre-coated with antigens followed by incubation with horse radish peroxidase (HRP)-conjugated anti-mouse IgG at 37 °C for 60 min. After washing, TMB substrate was used for development and the absorbance was read at 450 nm (minus 630 nm for wavelength correction) using the Synergy 2 Multi-Mode Microplate Reader (BioTek, Vermont).

### Measurement of serum ALT and AST

Levels of serum ALT and AST were quantified using commercialized ELISA kits according to the manuals (Nanjing Jiancheng Bioenginering Institute). Plates were read using Synergy 2 Multi-Mode Microplate Reader (BioTek, Vermont).

### Histopathological and immunohistochemical analysis

Liver tissues were collected and fixed in 4% paraformaldehyde (Sinopharm Chemical Reagent) and embedded in paraffin wax. 5-μm tissue sections were cut, dewaxed and rehydrated through xylene and alcohols and were subsequently subjected to hematoxylin and eosin (H&E) staining for histopathological assessment as previously described^26^. For Immunohistochemical (IHC) staining, tissue sections were dewaxed, rehydrated and antigen retrieved using proteinase K antigen retrieval solution (Abcam). Following this, tissue sections were washed three times with Tris-buffered saline (TBS) buffer and incubated with goat anti-Rat IgG (OriGene) as blocking reagent for 15 min. Intrahepatic expression of core antigen of HBV (HBc) was detected by incubating with anti-HBcAg monoclonal Ab (Gene Tech; GB058629) overnight at 4 °C. After washing with TBS buffer, tissue sections were incubated with biotinylated anti-rabbit IgG and streptavidin/horseradish peroxidase conjugates (ZSGB-Bio) for 20 min at 37 °C. DAB substrate was then added followed by counterstaining with hematoxylin. Finally, sections were dehydrated, cleared and mounted for analysis. Images were captured using Olympus BX46 microscope (Olympus, Tokyo, Japan).

### Quantification of HBV DNA and HBV RNA

Serum HBV DNA was measured by qPCR using an HBV DNA kit (Sansure Biotech). Intrahepatic HBV genomic DNA was extracted using a genomic DNA kit (Tiangen Biotech). Intrahepatic total RNA was extracted using TRIzol reagent (Invitrogen), and the RNA was reverse-transcribed into cDNA using a commercially available cDNA synthesis kit (CW Biotech). To distinguish the HBV cccDNA from the pAAV-HBV episome (plasmid DNA), the genomic DNA were treated by multi-enzyme digestion based the unique restriction site (SwaI) of pAAV-HBV1.2 plasmid as previously described^2^. Briefly, 1 μg of extracted DNA was digested with 10 U of SwaI restriction enzyme (New England Biolabs) for 15 min at 25 °C and then 1 U of ATP Dependent DNase (Takara Biomedical Technology) was added followed by incubation at 37 °C for 16 h. Real-time PCR for intrahepatic HBV DNA and RNA was performed by a Lightcycler^®^ 96 system (Roche) using the UltraSYBR mixture (CW Biotech). Sequences of the primers used are listed in Supplementary Table 1.

### Isolation of splenic mononuclear cells

Splenic mononuclear cells (MNCs) were isolated as previously described^20,22^. Briefly, spleen tissues were grinded gently and washed through a 200-μm sterile cell strainer. Cells were re-suspended in PBS and centrifuged at 400 *g* for 10 min. Cell pellets were next re-suspended with a suitable volume of Red Blood Cells (RBC) lysis buffer (Solarbio) for 5 min at 4 °C. Following this, 1× PBS was added to stop the RBC lysis and centrifuged at 400 g for 10 min to obtain splenic MNCs. After centrifugation, cells were re-suspended in RPMI-1640 medium containing 10% fetal bovine serum (FBS, BIOIND) and 1% penicillin-streptomycin (NCM Biotech) for subsequent in-vitro experiments.

### Isolation of hepatic mononuclear cells

Hepatic mononuclear cells (MNCs) were isolated as previously described^20,22^. Liver tissues were grinded gently and washed through a 200-μm sterile cell strainer. Cell suspensions were first centrifuged at 100 g for 1 min to precipitate and remove hepatocytes. Subsequently, cell supernatants were centrifuged at 400 g for 10 min and cell pellets were resuspended in 40% Percoll (GE Healthcare) followed by centrifugation at 800 g for 25 min. Cell pellets were next re-suspended with a suitable volume of Red Blood Cells (RBC) lysis buffer (Solarbio) for 5 min at 4 °C. Following this, 1× PBS was added to stop the RBC lysis and centrifuged at 400 g for 10 min to obtain hepatic MNCs. After centrifugation, cells were re-suspended in RPMI-1640 medium containing 10% fetal bovine serum (FBS, BIOIND) and 1% penicillin-streptomycin (NCM Biotech) for subsequent in-vitro experiments.

### Characterization of rAAV8-HBV1.3-transduced HBV-carrier mice

rAAV8-HBV1.3-transduced HBV-carrier mice were characterized by evaluating several aspects of T cell function as demonstrated in Supplementary Fig. 4. For the evaluation of cytokine-producing ability of T cells, 2 million hepatic MNCs were seeded per well into 96-well plates and incubated with 30 ng/mL PMA and 1 μg/mL ionomycin (both ordered from Beyotime) for 4 h in the presence of 5 μg/mL brefeldin A (Biolegend). Cytokine production of T cells was evaluated by surface and intracellular staining using Fixation/Permeabilization Solution Kit (BD Biosciences) according to the manuals. For the analysis of PD-1, LAG-3, and TIM-3 expression on T cells, hepatic and splenic MNCs were used and first incubated with Live/Dead Fixable Blue Dead Cells Stain Kit (Thermofisher) for 5 min. After washing, cells were incubated with antibody cocktails for 20 min at 4 °C in dark. Flow cytometry analysis was carried out on BD FACSymphony A3 (BD Biosciences). Data was analyzed using FlowJo V10.8 software (Tree Star). Fluorochrome-conjugated antibodies used in this study are listed in Supplementary Table 2.

### Analysis of antigen-specific memory B cell (MBC) response

Frequencies of HBsAg-specific MBCs were assessed by flow cytometry. For the preparation of HBsAg probes, HBsAg was first biotinylated using a Biotin Quick Labeling Kit (Friendbio Science) according to the manuals. Biotinylated HBsAg was next conjugated with Brilliant Violet 421-streptavidin (Biolegend) at a molar ratio of 4:1. Splenic MNCs were incubated with HBsAg probes for 20 min, and then stained with Live/Dead Fixable Blue Dead Cells Stain Kit (Thermofisher) for 5 min. After washing, cells were incubated with antibody cocktails for 20 minutes at 4 °C in dark. Flow cytometry analysis was carried out on BD FACSymphony A3 (BD Biosciences). Data was analyzed using FlowJo V10.8 software (Tree Star). Fluorochrome-conjugated antibodies used in this study are listed in Supplementary Table 2.

### Evaluation of antigen-specific T cell response

3 million splenic MNCs were seeded per well into 96-well U-bottom plates and incubated with HBsAg overlapping peptides (15-mers overlapping by 10 amino acids, GenScript Probio) at a concentration of 10 μg/mL for 16 h in the presence of 5 μg/mL brefeldin A (Biolegend). Splenic MNCs stimulated with 2 μg/mL staphylococcal enterotoxin B (SEB, Creative Diagnostics) were used as positive control. Cytokine production of antigen-specific T cells was evaluated by surface and intracellular staining using Fixation/Permeabilization Solution Kit (BD Biosciences) according to the manuals. Frequencies of cytokine-producing T cells were determined by FACS analysis. Background cytokine staining was subtracted, as defined by staining in the samples incubated with medium alone. Flow cytometry analysis was carried out on BD FACSymphony A3 (BD Biosciences). Data was analyzed using FlowJo V10.8 software (Tree Star). Fluorochrome-conjugated antibodies used in this study are listed in Supplementary Table 2.

### Statistical analysis

Statistical analyses were performed using GraphPad Prism software (v6.0; GraphPad Software, La Jolla, CA, USA). An unpaired, two-tailed Student’s *t* test was applied for comparisons between two groups, and differences among multiple groups were analyzed by two-way analysis of variance. A *p* value less than 0.05 was considered statistically significant (*p ≤ 0.05, **p ≤ 0.01, ***p ≤ 0.001).

### Reporting summary

Further information on research design is available in the Nature Research Reporting Summary linked to this article.

### Supplementary information

Supplemental Information

Reporting Summary

## Data Availability

All data are available upon reasonable request to the corresponding authors.
