# Supplementary material for: A therapeutic hepatitis B mRNA vaccine with strong immunogenicity and persistent virological suppression
Source: NPJ Vaccines. 2024 Feb 3;9:22. doi: 10.1038/s41541-024-00813-3 (PMC10838333; doi:10.1038/s41541-024-00813-3)
Supplement: Supplementary file 1 — Supplemental Information [file 41541_2024_813_MOESM1_ESM.pdf]

Supplementary Information for

A therapeutic hepatitis B mRNA vaccine with strong immunogenicity and persistent virological suppression

Huajun Zhao[1#✉], Xianyu Shao[2,3#], Yating Yu[1#], Lulu Huang[2,3#], Narh Philip Amor[2,3#], Kun Guo[2,3], Changzhen Weng[2,3], Weijun Zhao[2,3], Ailu Yang[1], Jiesen Hu[4], Hongbao Yang[3], Zhenguang Liu[5], Qiuju Han[1], Leilei Shi[6], Shiyu Sun[7], Jian Zhang[1✉], Ang Lin[2,3✉], Yong Yang[2,3,8✉]

Affiliations:

1. Institute of Immunopharmaceutical Sciences, Key Laboratory of Chemical Biology, School of Pharmaceutical Sciences, Shandong University, Jinan, Shandong, China.
2. Vaccine Center, School of Basic Medicine and Clinical Pharmacy, China Pharmaceutical University, Nanjing, China.
3. Center for New Drug Safety Evaluation and Research, China Pharmaceutical University, Nanjing, China.
4. Firestone Biotechnologies, Shanghai, China.
5. College of Veterinary Medicine, Nanjing Agricultural University, Nanjing, China.
6. Precision Research Center for Refractory Diseases in Shanghai General Hospital, Shanghai Jiao Tong University, Shanghai, China.
7. Key Laboratory of Infection and Immunity, Institute of Biophysics, Chinese Academy of Sciences, Beijing, China.
8. School of Pharmacy, Xuzhou Medical University, Xuzhou, Jiangsu 221004, P.R. China.

#Authors contributed equally

Lead contact:

Yong Yang, yy@cpu.edu.cn; Ang Lin, anglin@cpu.edu.cn

Supplementary Figure 1

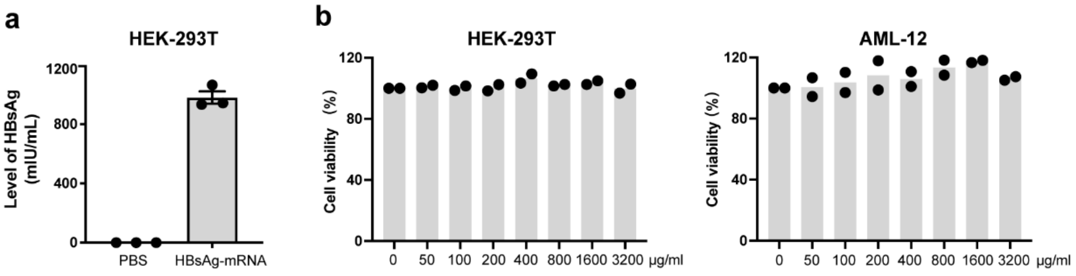

Supplementary Figure 1. Translation of HBsAg-encoding mRNA and cytotoxicity of HBV mRNA vaccine.

a HEK-293T cells transfected with 2μg m1Ψ-modified HBsAg-encoding mRNAs were harvested 48 hours after transfection. HBsAg levels in cell lysates were detected. Data compiled from three independent experiments are shown as mean ± SEM. **b** HEK-293T and AML-12 cells were treated with escalating doses of HBV mRNA vaccines for 24 hours. Cell viability was determined with CCK8 assays and depicted as percentage relative to untreated cells. Data from two independent experiments are shown.

Supplementary Figure 2

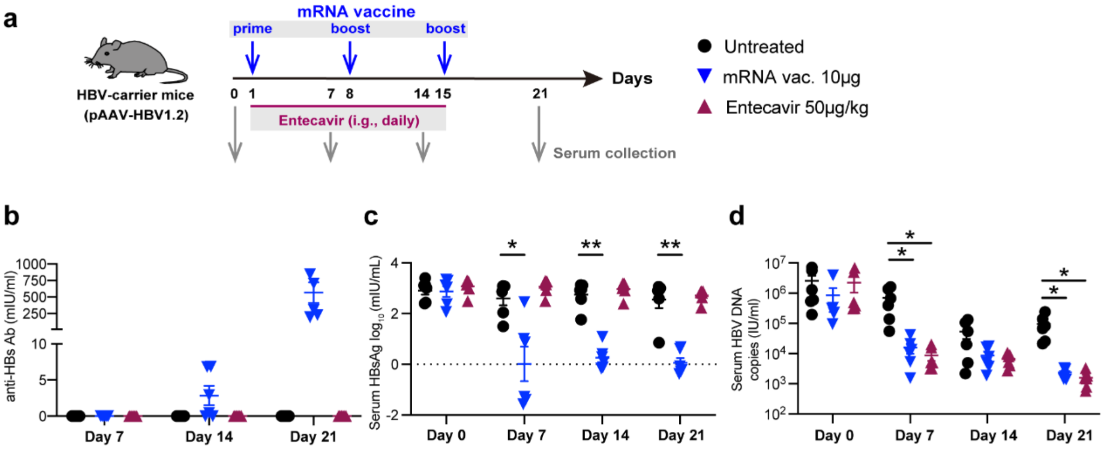

Supplementary Figure 2. HBV mRNA vaccine showed superior efficacy over Entecavir.

a pAAV/HBV1.2-transduced HBV-carrier mice (n=6/group) were either immunized i.m. with 10μg HBV mRNA vaccines three times at a 1-week interval or were administered with Entecavir (ETV, 50μg/kg) via oral gavage for 15 days consecutively. HBV-carrier mice administered with PBS were used as control. Sera samples were collected at the indicated time points. **b** Levels of anti-HBs Abs were evaluated. **c** Serum HBsAg was measured by chemiluminescence immunoassay (CLIA). **d** Serum HBV DNA was quantified. Two-way ANOVA was used for statistical analysis. Data are shown as Mean ± SEM. $*p \leq 0.05$, $**p \leq 0.01$.

Supplementary Figure 3

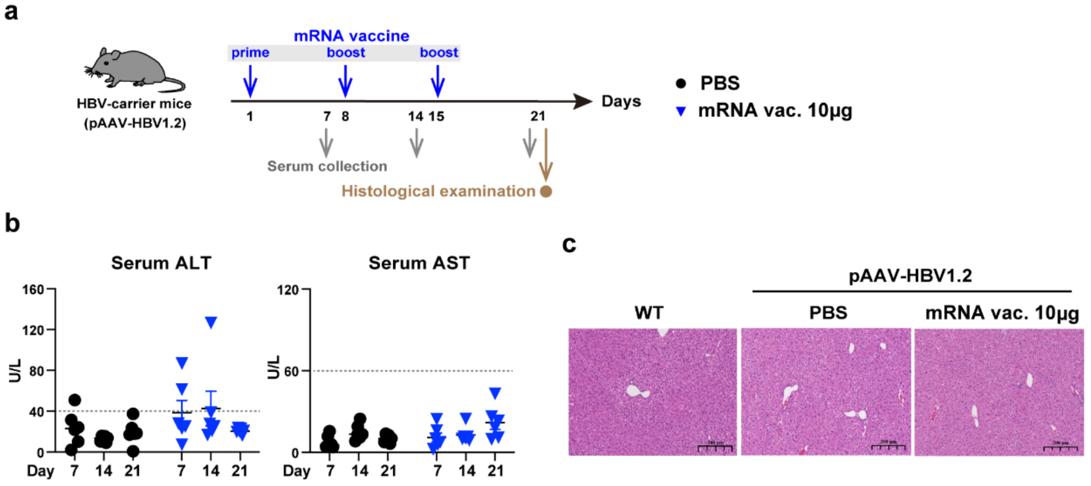

Supplementary Figure 3. HBV mRNA vaccine induced very limited hepatotoxicity and liver injury.

a pAAV/HBV1.2-transduced HBV-carrier mice (n=6/group) were immunized i.m. with 10μg HBV mRNA vaccines three times at a 1-week interval. Sera samples and liver tissues were collected at the indicated time points. **b** Levels of serum ALT and AST were assessed. Data are shown as Mean ± SEM. The dotted line depicts reported normal levels of the two parameters. **c** Hematoxylin and eosin (H&E) staining for histopathologic analysis of liver tissues collected at the indicated time point. Scale bar: 200 μm; WT: wide-type healthy mice.

Supplementary Figure 4

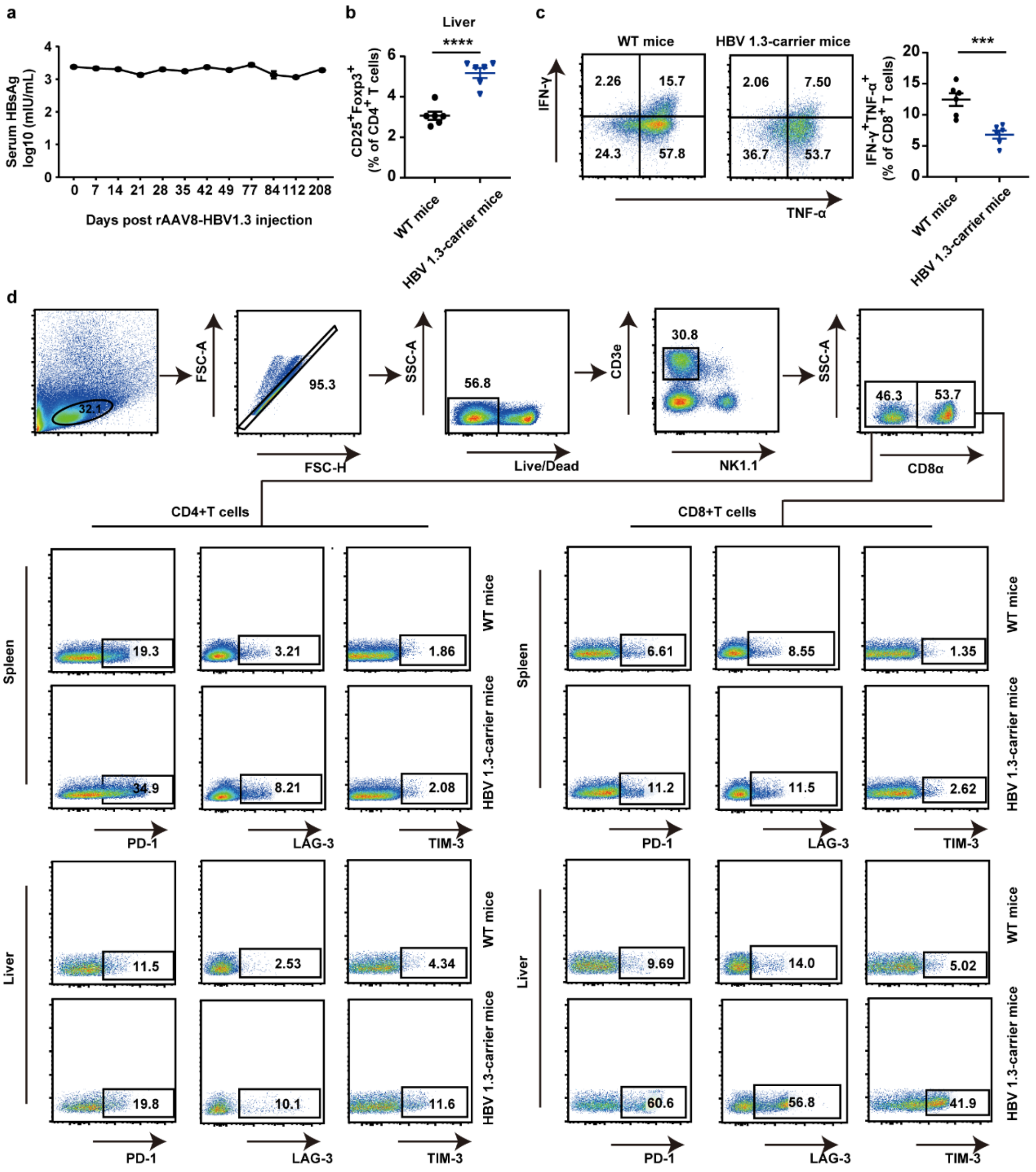

Supplementary Figure 4. rAAV8-HBV1.3-transduced mice showed persistent HBV viremia and immunotolerance.

a C57BL/6J mice (n=6) were intravenously injected with 1 × 10^{10} vector genome equivalent of rAAV8-HBV1.3. Serum HBsAg levels were detected at the indicated time points after rAAV8-HBV1.3 injection. **b** Frequencies of hepatic $CD4^{+}CD25^{+}Foxp3^{+}$ cells from rAAV8-HBV1.3 mice and WT mice are shown. **c** Hepatic MNCs (2 × 10^{6}) from rAAV8-HBV1.3 mice and WT mice were stimulated with PMA/ionomycin in vitro for 4 hours in the presence of brefeldin A (5μg/mL), and the production of IFN-γ

and TNF-α by $CD8^+$ T cells was analyzed by flow cytometry. **d** Expression of PD-1, LAG-3, and TIM-3 on hepatic and splenic $CD4^+$ and $CD8^+$ T cells from rAAV8-HBV1.3 mice and WT mice were analyzed by flow cytometry. An unpaired, two-tailed Student's *t* test was used for statistical analysis. ***$p \leq 0.001$, ****$p \leq 0.0001$.

Supplementary Figure 5

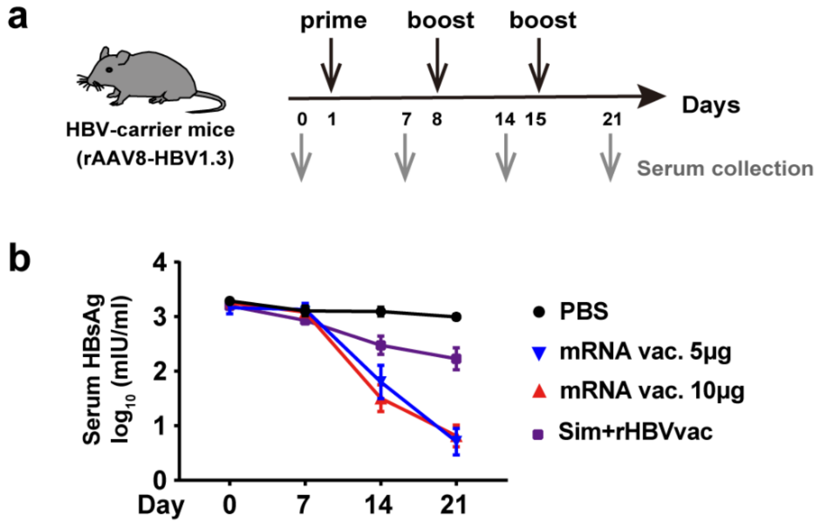

Supplementary Figure 5. HBV mRNA vaccine showed superior efficacy over recombinant therapeutic vaccine in viral clearance.

a rAAV/HBV1.3-transduced HBV-carrier mice (n=6/group) were immunized i.m. with HBV mRNA vaccines or recombinant HBV therapeutic vaccines ("Sim+rHBV") three times at a 1-week interval. HBV-carrier mice administered with PBS were used as control. Sera samples were collected at the indicated time points. **b** Serum HBsAg levels were detected by chemiluminescence immunoassay (CLIA). Data are shown as Mean ± SEM.

Supplementary Figure 6

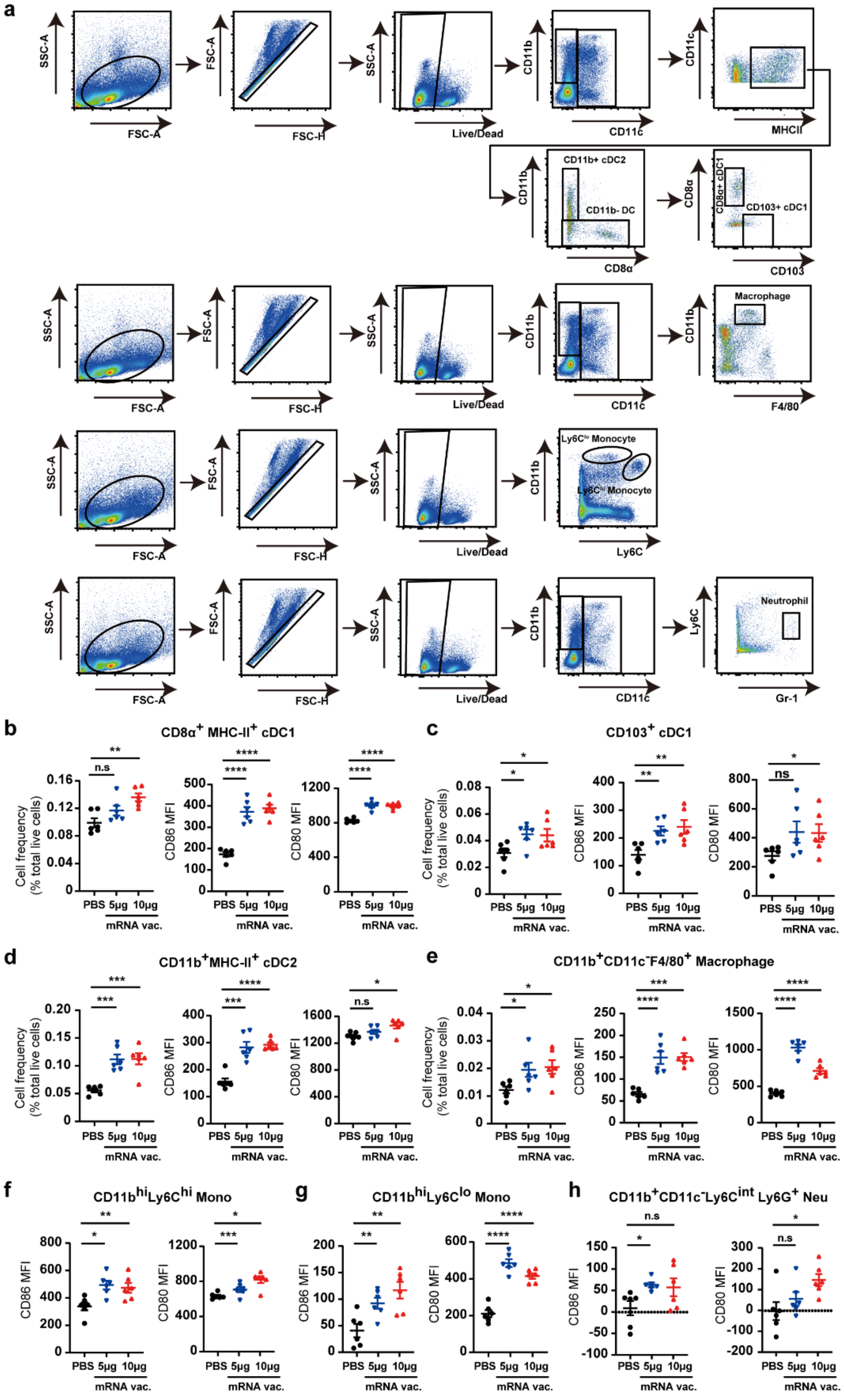

Supplementary Figure 6. HBV mRNA vaccine induced strong innate immune activation in HBV-carrier mice.

rAAV/HBV1.3-transduced HBV-carrier mice (n=6/group) were i.m. injected with 5μg

or 10μg HBV mRNA vaccine. Splenic mononuclear cells (MNCs) were collected 12 hours after immunization. **a** Gating strategy for phenotypic identification of the indicated innate immune cell subsets. **b-e** Frequencies of $CD8\alpha^+$ cDC1s, $CD103^+$ cDC1s, $CD11b^+$ cDC2s, and $F4/80^+$ Macrophages and expression of CD80 and CD86 on these cell subsets were evaluated by flow cytometry. Mean fluorescence intensity (MFI) values are shown. **f-h** Expression of CD80 and CD86 on $CD11b^{hi}Ly6C^{lo}$ monocytes, $CD11b^{hi}Ly6C^{hi}$ monocytes and $CD11b^+CD11c^-Ly6C^{int}Ly6G^+$ neutrophils were analyzed by flow cytometry. MFI values are shown. An unpaired, two-tailed Student's *t* test was used for statistical analysis. $*p \le 0.05$, $**p \le 0.01$, $***p \le 0.001$, $****p \le 0.0001$.

Supplementary Figure 7

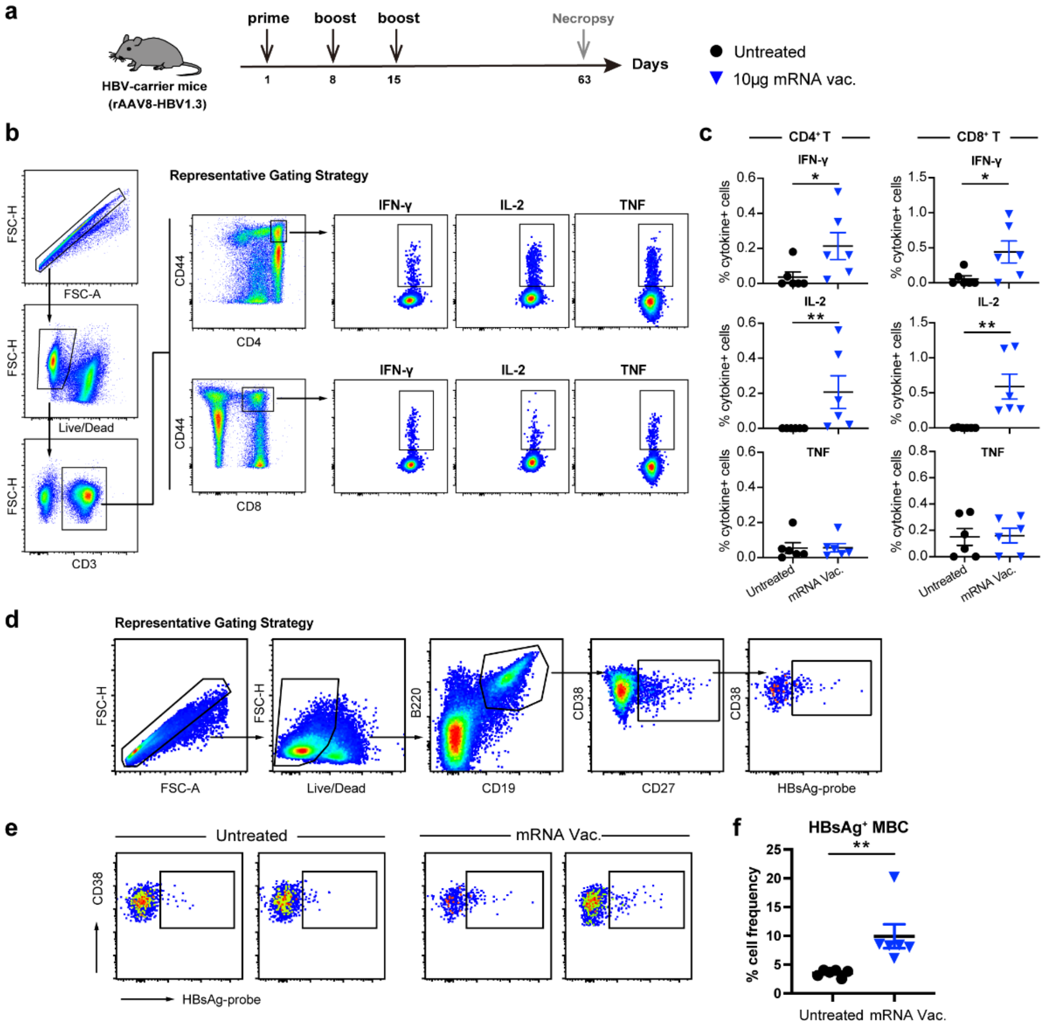

Supplementary Figure 7. HBV mRNA vaccine induced robust HBsAg-specific Th1-biased T cell and memory B cell responses.

a rAAV/HBV1.3-transduced HBV-carrier mice (n=6/group) were immunized i.m. with 10μg HBV mRNA vaccines three times at a 1-week interval. HBV-carrier mice administered with PBS were used as control. Spleens were collected 48 days after the 3rd vaccine dose. **b-c** Splenic cells were stimulated with 10μg/ml HBsAg overlapping peptides (15-mers overlapping by 10 amino acids) for 16 hours in the presence of brefeldin A. Cells stimulated with staphylococcal Enterotoxin B (SEB) were used as positive control. **b** Representative gating of SEB-stimulated cells secreting cytokines. **c** Quantification of cytokine-producing antigen-specific CD4^{+} and CD8^{+} T cells following overlapping peptides stimulation. **d-f** Frequencies of HBsAg-specific

memory B cells (MBCs) in spleens were evaluated. **d** Representative gating of HBsAg-specific MBCs. **e** Data from two representative animals from each group are shown. **f** Frequencies of HBsAg-specific MBCs in spleens of mice from the indicated groups. An unpaired, two-tailed Student's *t* test was used for statistical analysis. Data are shown as Mean ± SEM. $^{*}p \leq 0.05$, $^{**}p \leq 0.01$.

Supplementary Tables

Supplementary Table 1. List of primers for HBV DNA and RNA quantification.

Reagent	Primers
mouse-GAPDH-F	5'-AGG TCG GTG TGA ACG GAT TTG-3'
mouse-GAPDH-R	5'-TGT AGA CCA TGT AGT TGA GGT CA-3'
HBV-cccDNA-F	5'-CGT CTG TGC CTT CTC ATC TGC-3'
HBV-cccDNA-R	5'-GCA CAG CTT GGA GGC TTG AA-3'
HBV-DNA-F	5'-CAC ATC AGG ATT CCT AGG ACC-3'
HBV-DNA-R	5'-GGT GAGTGA TTG GAG GTT G-3'
HBV-total-RNA-F	5'-TCA CCA GCA CCA TGC AAC-3'
HBV-total-RNA-R	5'-AAG CCA CCC AAG GCA CAG-3
HBV-3.5kb-RNA-F	5'-GAG TGT GGA TTC GCACTC C-3'
HBV-3.5kb-RNA-R	5'-GAG GCG AGG GAG TTC TTC T-3'

Supplementary Table 2. List of anti-mouse antibodies used for FACS analysis.

Antibody	Clone	Manufacturer	Cat. Number	Dilution
CD45R/B220	RA3-6B2	Biolegend	103236	1:100
CD19	6D5	Biolegend	115508	1:200
CD38	90	Biolegend	102730	1:200
CD27	LG.3A10	Biolegend	124241	1:200
CD3	17A2	Biolegend	100204	1:100
CD4	GK1.5	Biolegend	100414	1:100
CD44	IM7	Biolegend	103024	1:200
IFN-γ	XMG1.2	Biolegend	505830	1:200
TNF	MP6-XT22	Biolegend	506329	1:200
IL-2	JES6-5H4	Biolegend	503840	1:100
PD-1	29F.1A12	Biolegend	135210	1:200
LAG-3	C9B7W	Biolegend	125221	1:200
TIM-3	RMT3-23	Biolegend	119725	1:200
CD103	2E7	Biolegend	121432	1:200
CD11c	N418	Biolegend	117320	1:200
MHC-II	M5/114.15.2	Biolegend	107645	1:200
CD80	16-10A1	Biolegend	104733	1:200
CD86	IT2.2	Biolegend	105036	1:200
NK1.1	PK136	Biolegend	108752	1:200
Gr-1	RB6-8C5	eBioscience	17-5931-82	1:500
Ly6C	HK1.4	eBioscience	12-5932-82	1:200
CD11b	M1/70	eBioscience	11-0112-85	1:100
CD25	PC61.5	eBioscience	17-0251-82	1:200
Foxp3	FJK-16s	eBioscience	45-5773-82	1:100
F4/80	BM8	eBioscience	45-4801-82	1:100
CD8α	53-6.7	BD	562283	1:200
