# Supplementary material for: A therapeutic hepatitis B mRNA vaccine with strong immunogenicity and persistent virological suppression
Source: NPJ Vaccines. 2024 Feb 3;9:22. doi: 10.1038/s41541-024-00813-3 (PMC10838333; doi:10.1038/s41541-024-00813-3)
Supplement: Supplementary file 2 — Reporting Summary [file 41541_2024_813_MOESM2_ESM.pdf]

nature portfolio

Corresponding author(s): Yong Yang, Ang Lin, Jiang Zhang, Huajun Zhao

Last updated by author(s): Jan 5, 2024

Reporting Summary

Nature Portfolio wishes to improve the reproducibility of the work that we publish. This form provides structure for consistency and transparency in reporting. For further information on Nature Portfolio policies, see our Editorial Policies and the Editorial Policy Checklist.

Statistics

For all statistical analyses, confirm that the following items are present in the figure legend, table legend, main text, or Methods section.

n/a	Confirmed	
☐	☒	The exact sample size (*n*) for each experimental group/condition, given as a discrete number and unit of measurement
☐	☒	A statement on whether measurements were taken from distinct samples or whether the same sample was measured repeatedly
☐	☒	The statistical test(s) used AND whether they are one- or two-sided<br>*Only common tests should be described solely by name; describe more complex techniques in the Methods section.*
☐	☒	A description of all covariates tested
☐	☒	A description of any assumptions or corrections, such as tests of normality and adjustment for multiple comparisons
☐	☒	A full description of the statistical parameters including central tendency (e.g. means) or other basic estimates (e.g. regression coefficient) AND variation (e.g. standard deviation) or associated estimates of uncertainty (e.g. confidence intervals)
☐	☒	For null hypothesis testing, the test statistic (e.g. *F*, *t*, *r*) with confidence intervals, effect sizes, degrees of freedom and *P* value noted<br>*Give P values as exact values whenever suitable.*
☒	☐	For Bayesian analysis, information on the choice of priors and Markov chain Monte Carlo settings
☒	☐	For hierarchical and complex designs, identification of the appropriate level for tests and full reporting of outcomes
☒	☐	Estimates of effect sizes (e.g. Cohen's *d*, Pearson's *r*), indicating how they were calculated

Our web collection on statistics for biologists contains articles on many of the points above.

Software and code

Policy information about availability of computer code

Data collection	FACS Diva software in our study was commercial.
Data analysis	FlowJo software V10.8 in our study was commercial.

For manuscripts utilizing custom algorithms or software that are central to the research but not yet described in published literature, software must be made available to editors and reviewers. We strongly encourage code deposition in a community repository (e.g. GitHub). See the Nature Portfolio guidelines for submitting code & software for further information.

Data

Policy information about availability of data

All manuscripts must include a data availability statement. This statement should provide the following information, where applicable:

- Accession codes, unique identifiers, or web links for publicly available datasets
- A description of any restrictions on data availability
- For clinical datasets or third party data, please ensure that the statement adheres to our policy

All data are available upon reasonable request to the corresponding authors.

Research involving human participants, their data, or biological material

Policy information about studies with human participants or human data. See also policy information about sex, gender (identity/presentation), and sexual orientation and race, ethnicity and racism.

Reporting on sex and gender	Our study don't invole human participants or human data.
Reporting on race, ethnicity, or other socially relevant groupings	Our study don't involve human participants or human data.
Population characteristics	Our study don't involve human participants or human data.
Recruitment	Our study don't involve human participants or human data.
Ethics oversight	Our study don't involve human participants or human data.

Note that full information on the approval of the study protocol must also be provided in the manuscript.

Field-specific reporting

Please select the one below that is the best fit for your research. If you are not sure, read the appropriate sections before making your selection.

☒ Life sciences ☐ Behavioural & social sciences ☐ Ecological, evolutionary & environmental sciences

For a reference copy of the document with all sections, see nature.com/documents/nr-reporting-summary-flat.pdf

Life sciences study design

All studies must disclose on these points even when the disclosure is negative.

Sample size	We used 5 to 6 animals in each group, and unpaired student's t test or two-way analysis of variance was applied in our study.
Data exclusions	No data were excluded from the analysis.
Replication	All attempts at replication were successful.
Randomization	Samples were randomized into experimental groups.
Blinding	None

Reporting for specific materials, systems and methods

We require information from authors about some types of materials, experimental systems and methods used in many studies. Here, indicate whether each material, system or method listed is relevant to your study. If you are not sure if a list item applies to your research, read the appropriate section before selecting a response.

Materials & experimental systems

n/a	Involved in the study	
☐	☒	Antibodies
☐	☒	Eukaryotic cell lines
☒	☐	Palaeontology and archaeology
☐	☒	Animals and other organisms
☒	☐	Clinical data
☒	☐	Dual use research of concern
☒	☐	Plants

Methods

n/a	Involved in the study	
☒	☐	ChIP-seq
☐	☒	Flow cytometry
☒	☐	MRI-based neuroimaging

Antibodies

Antibodies used	anti-mouse CD45R/B220 (clone: RA3-6B2), CD19 (clone: 6D5), CD38 (clone: RA3-6B2), CD27 (clone: 90), CD3 (clone: LG.3A10),CD4 (clone: 17A2),CD44 (clone: GK1.5), CD62L (clone: IM7), IFN-γ (clone: XMG1.2)? TNF-α (clone: MP6-XT22)? IL-2 (clone: JES6-5H4), PD-1 (clone: 29F.1A12), LAG-3 (clone: C9B7W), TIM-3 (clone: RMT3-23), NK1.1(clone: PK136), CD103(clone: 2E7), CD11c(clone:

	N418)?MHCII(clone: M5/114.15.2)? CD80(clone:16-10A1), CD86(clone: IT2.2), Gr-1(clone: RB6-8C5), LY6C(clone: HK1.4), CD11b(clone: M1/70)? F4/80(clone: BM8), CD8α(clone: 53-6.7).
Validation	Anti-mouse monoclonal used for FACS analysis were from biolegend, BD and ebioscience according to the manufacturers' instructions.

Eukaryotic cell lines

Policy information about cell lines and Sex and Gender in Research

Cell line source(s)	HEK 293T and AML12 cells were purchased from ATCC.
Authentication	All cell lines were tested for mycoplasma contamination.
Mycoplasma contamination	All cell lines were tested negative for mycoplasma contamination.
Commonly misidentified lines (See ICLAC register)	None

Animals and other research organisms

Policy information about studies involving animals; ARRIVE guidelines recommended for reporting animal research, and Sex and Gender in Research

Laboratory animals	C57BL/6J mice (5-6weeks old) was purchased from Beijing HFK Bioscience Co.Ltd.
Wild animals	C57BL/6J mice (5-6weeks old) was purchased from Beijing HFK Bioscience Co.Ltd.
Reporting on sex	We only used male mice to generate the HBV-carrier mice model.
Field-collected samples	The study didn't involve samples collected from the field.
Ethics oversight	The Institutional Animal Care and Use Committee of Shandong University

Note that full information on the approval of the study protocol must also be provided in the manuscript.

Plants

Seed stocks	Our study didn't involve plants data.
Novel plant genotypes	Our study didn't involve plants data.
Authentication	Our study didn't involve plants data.

Flow Cytometry

Plots

Confirm that:

☒ The axis labels state the marker and fluorochrome used (e.g. CD4-FITC).

☒ The axis scales are clearly visible. Include numbers along axes only for bottom left plot of group (a 'group' is an analysis of identical markers).

☒ All plots are contour plots with outliers or pseudocolor plots.

☒ A numerical value for number of cells or percentage (with statistics) is provided.

Methodology

Sample preparation	Hepatic and splenic mononuclear cell from C57BL/6J mice were isolated by centrifugation, and detailed procedures are available in the Methods section.
Instrument	Data were collected using a BD FACSymphony A3.

Software	FlowJo software (Tree Star).
Cell population abundance	Our study didn't involve cell sorting procedure.
Gating strategy	The gating strategy was provided in the supplementary figures.

☒ Tick this box to confirm that a figure exemplifying the gating strategy is provided in the Supplementary Information.
